# Mycelial biomass estimation and metabolic quotient of *Lentinula edodes* using species-specific qPCR

**DOI:** 10.1371/journal.pone.0232049

**Published:** 2020-05-18

**Authors:** Mayuko Jomura, Tomoko Kuwayama, Yuto Soma, Muneyoshi Yamaguchi, Masabumi Komatsu, Yutaka Maruyama

**Affiliations:** 1 Department of Forest Science and Resources, College of Bioresource Sciences, Nihon University, Fujisawa, Kanagawa, Japan; 2 Forestry and Forest Products Research Institute, Tsukuba, Ibaraki, Japan; USDA Forest Service, UNITED STATES

## Abstract

*Lentinula edodes*, commonly known as shiitake, is an edible mushroom that is cultivated and consumed around the globe, especially in Asia. Monitoring mycelial growth inside a woody substrate is difficult, but it is essential for effective management of mushroom cultivation. Mycelial biomass also affects the rate of wood decomposition under natural conditions and must be known to determine the metabolic quotient, an important ecophysiological parameter of fungal growth. Therefore, developing a method to measure it inside a substrate would be very useful. In this study, as the first step in understanding species-specific rates of fungal decomposition of wood, we developed species-specific primers and qPCR procedures for *L*. *edodes*. We tested primer specificity using strains of *L*. *edodes* from Japan and Southeast Asia, as well as related species of fungi and plant species for cultivation of *L*. *edodes*, and generated a calibration curve for quantification of mycelial biomass in wood dust inoculated with *L*. *edodes*. The qPCR procedure we developed can specifically detect *L*. *edodes* and allowed us to quantify the increase in *L*. *edodes* biomass in wood dust substrate and calculate the metabolic quotient based on the mycelial biomass and respiration rate. Development of a species-specific method for biomass quantification will be useful for both estimation of mycelial biomass and determining the kinetics of fungal growth in decomposition processes.

## Introduction

*Lentinula edodes* (Berk.) Pegler (shiitake) is the second most cultivated species of mushroom in the world after the white button mushroom, *Agaricus bisporus* (Lange) Imbach. *Lentinula edodes* is a saprotrophic fungus with a distribution in eastern Asia [1, 2] and many strains and cultivars are in commercial use. Most of the *L*. *edodes* mushrooms sold in markets are cultivated, not collected. Sawdust cultures mixed with nitrogen-rich substrates produce fruiting bodies in 6 months or less owing to the ideal nutritional and environmental conditions for fungal growth. Bed logs, usually *Quercus* spp., produce higher quality fruiting bodies than the former method, but only emerge at least 2 years after inoculation of fungal mycelia because of the lower nutrient supply and more variable environmental conditions of cultivation. Both production methods pose difficulties for optimizing the conditions for mycelial growth and development of fruiting bodies, and mushroom producers must manage the quality, quantity, and intensity of mushroom production based mostly on experience, as little evidence is available on optimal cultivation conditions. Developing cultivation methods based on fungal growth would enable efficient production of fruiting bodies and overcome the recent difficulties in mushroom production posed by the high temperatures and changes in precipitation patterns caused by climate change [3].

One of the major indicators of hyphal growth used in the cultivation of *L*. *edodes* is fungal biomass in the substrate. Because *L*. *edodes* is a white-rot fungus, lignin degradation results in whitening of the substrate, and visually detected mycelial abundance is usually used to determine the growth rate and maturity of the fungus. However, in order to monitor fungal growth more accurately, a tool for quantification of fungal biomass is useful. Several methods have been introduced for estimation of fungal biomass in woody substrates, such as measurement of ergosterol and chitin, components of the fungal cell membrane and cell wall, respectively [4, 5]. Quantitative PCR (qPCR) using universal primers is also used to detect DNA copy number of fungi in a target substrate as an index of abundance [4]. However, all of these methods vary widely among species in their usefulness for quantification of biomass because the internal transcribed spacer copy number varies between fungal species and the ratio of gene copy number to fungal biomass varies among species and life cycle stages (i.e., between dikaryotic and monokaryotic mycelia) [4]. Development of species-specific primers would permit accurate biomass estimation by using qPCR, and this can be done for species with available sequence data. Species-specific qPCR has been used for microbial ecology approaches, such as for estimating the biomass distribution in soil of *Tricholoma matsutake* (S. Ito et Imai) Sing. (matsutake), a prized edible mycorrhizal fungus, and for examining interspecific competition between two fungal species [6, 7]. Therefore, development of species-specific primers for qPCR of *L*. *edodes* in a woody substrate would be a useful tool for measuring fungal biomass to improve cultivation efficiency.

Methods for species-specific estimation of microbial biomass would also be useful to investigate microbial ecology and physiology in natural conditions, which are strongly linked to the global carbon cycle. Fungal biomass is one of the factors that explains the variation in the decomposition rate of wood at the local scale [8], and metagenomic analysis of dead wood in forests shows that a few fungal species account for more than 90% of the relative sequence abundance in a unit of dead wood [9, 10]. The decomposition rate of wood varies widely with the fungal species inoculated [11]. Species-level biomass estimation tools would clarify spatial and temporal colonization patterns of dominant fungal species and aid in detecting differences in decomposition rate between fungi. The microbial metabolic quotient (MMQ) is defined as the quantity of carbon respired per unit of microbial biomass, which is a fungal physiological parameter that responds to environmental factors [12–18]. Species-level MMQ would be useful to clarify the response of metabolic activity at the species level to environmental factors. Therefore, tools for species-specific fungal biomass estimation would be useful for evaluation of decomposition processes in cultivated and natural conditions.

In this study, to quantify the biomass of a single species of fungi in a woody substrate, we developed a species-specific qPCR method, using *L*. *edodes* as a model. We designed a set of primers based on published sequence data [19], examined primer specificity using DNA of strains of *L*. *edodes* collected in Japan and Southeast Asia, as well as related fungi and plants used for cultivation of *L*. *edodes*, and produced a standard curve for estimation of fungal biomass in a woody substrate. Finally, we inoculated wood dust with *L*. *edodes* and measured biomass and species-specific MMQ.

## Materials and methods

### Fungal and plant materials used for testing primer specificity

Table 1 lists the fungi used to test primer specificity, including *L*. *edodes* strains (from Japan and Southeast Asia), fungi closely related to *L*. *edodes*, commercial saprotrophic fungi, pathogenic fungi present in commercial cultivation settings, and plants used for commercial cultivation. All fungal species were grown at 25°C on potato dextrose agar (Nissui, Tokyo, Japan) plates for 2 weeks. Mycelia and plant materials were frozen in liquid nitrogen and lyophilized using a deep-freeze-drying vacuum pump system (Tsukuba Origo Service, Ushiku, Japan) for 48 h. Lyophilized pure cultures of mycelia and plant materials were ground to powder with a Multi-beads Shocker (Yasui Kikai Corporation, Osaka, Japan). Extracted DNA was used for PCR and qPCR.

**Table 1:** Fungal strains and plant materials used in this study.

Group	Species	type	Source or reference*^1^	Sample No.
*Lentinus edodes*	*Lentinus edodes* (Berk.) Sing.	domestic	FMC66	1
	*Lentinus edodes* (Berk.) Sing.	domestic	FMC115	2
	*Lentinus edodes* (Berk.) Sing.	domestic	FMC390	3
	*Lentinus edodes* (Berk.) Sing.	domestic	FMC392	4
	*Lentinus edodes* (Berk.) Sing.	domestic	FMC474	5
	*Lentinus edodes* (Berk.) Sing.	foreign	FMC492	6
	*Lentinus edodes* (Berk.) Sing.	foreign	FMC48	7
	*Lentinus edodes* (Berk.) Sing.	foreign	FMC50	8
	*Lentinus edodes* (Berk.) Sing.	foreign	FMC51	9
Related species	*Lampteromyces japonicus* (Kawam.) Sing.		FMC357	10
	*Collybia peronata* (Bolt.:Fr.) Kummer		FMC614	11
	*Collybia butyracea* (Bull.:Fr.) Quel.		FMC611	12
	*Collybia cookei* (Bres.) J.D.Arnold		FMC613	13
	*Marasmius purpureostriatus* Hongo		FMC619	14
	*Marasmius siccus* (Schw.) Fr.		FMC620	15
	*Crinipellis stipitaria* (Fries) Patouillard		FMC656	16
	*Lentinus boryanus* (Berkeley & Montagne) Singer		FMC670	17
	*Gymnopus subsulphurea*		WD2410	18
Commercial species	*Flammulina velutipes* (Curt.: Fr.) Sing.		FMC223	19
	*Pleurotus ostreatus* (Jacq.:Fr.) Kummer		FMC235	20
	*Pholiota nameko* (T.Ito) S. Ito & Imai in Imai		FMC260	21
	*Pholiota adiposa* (Fr.) Kummer		FMC299	22
	*Naematoloma sublateritium* (Fr.) Karst		FMC303	23
	*Agaricus bisporus* (J. Lange) Imbach		FMC306	24
	*Grifola frondosa* (Dicks.: Fr.) S.F. Gray		FMC318	25
	*Panellus serotinus* (Pers.:Fr.) Kuhn.		FMC494	26
	*Lyophyllum shimeji* (Kawam.) Hongo		FMC516	27
	*Pleurotus pulmonarius* (Fr.) Quel.		FMC571	28
	*Panellus stipticus* (Bull.) P. Karst.		WD162	29
Saprotrophs	*Steccherinum ochraceum* (Pers.) S. F. Gray		FMC316	30
	*Coriolus versicolor* (L.: Fr.) Quel.		FMC334	31
	*Schizophyllum commune* Fr.: Fr.		WD61	32
	*Porodisculus orientalis* J.S. Lee & H.S. Jung = Porodisculus pendulus ss. Imazeki		WD649	33
	*Pycnoporus coccineus* (Fr.) Bondartsev & Singer		WD866	34
	*Pycnoporus coccineus* (Fr.) Bondartsev & Singer		WD2263	35
	*Trametes hirsuta* (Wulfen) Pilát f. hirsuta		WD1567	36
	*Microporus affinis* (Blume & T. Nees) Kuntze		WD1713	37
	*Lenzites betulinus* (L.) Fr.		WD1987	38
	*Perenniporia fraxinea* (Bull.) Ryvarden		WD672	39
	*Daedalea dickinsii* Yasuda		WD1309	40
	*Inonotus xeranticus* (Berk.) Imazeki & Aoshima		WD891	41
	*Steccherinum rhois* (Schwein.) Banker		WD2004	42
Destructive species	*Trichoderma harzianum* Rifai		KRCF131	43
	*Penicillium brevicompactum* C. Ramírez		KRCF182	44
	*Penicillium fellutanum* S. Abe		KRCF200	45
	*Trichoderma atroviride* Bissett		KRCF222	46
	*Trichoderma pseudokoningii* Rifai		KRCF305	47
	*Trichoderma longibrachiatum* Rifai		KRCF306	48
	*Aspergillus ochraceus* Kral ex Blumentritt		KRCF324	49
	*Aspergillus fumigatus* Kral ex Blumentritt		KRCF335	50
	*Rhizopus sp*.		KRCF337	51
	*Mucor sp*.		KRCF498	52
	*Hypocrea lactea* (Fr.) Fr.		KRCF1150	53
	*Hypocrea peltata* Berk.		KRCF1158	54
	*Trichoderma harzianum* Rifai		WD1507	55
	*Trichoderma harzianum* Rifai		WD1508	56
Plant materals	*Castanea crenata* Siebold et Zucc.	leaf		57
	*Quercus serrata* Murray	leaf		58
	*Quercus acutissima* Carruth.	leaf		59
	*Quercus crispula* Blume	leaf		60
	*Fagus crenata* Blume	leaf		61
	*Cryptomeria japonica* (L.f.) D.Don	leaf		62
	*Zea mays* L.			63
	*Triticum aestivum* L.			64
	*Fagus crenata* Blume	sawdust		65
	*Oryza sativa* L.	* *		66

*1FMC:from Dr. Masabumi Komatsu, Forestry and Forest Products Research Institute, KRCF: from Kazuhiro Miyazaki, Kyusyu Research Center, Forestry and Forest Products Research Institute, WD: from Yuko Oota, Forestry and Forest Products Research Institute

To quantify *L*. *edodes* biomass, we needed to scale up *L*. *edodes* mycelia to quantities of micrograms to make a dilution series. However, such small quantities of powdered mycelia were difficult to measure using an electronic precision balance. Therefore, we diluted the powdery *L*. *edodes* mycelia with powdered *Pleurotus pulmonarius* mycelia and fine sawdust to make the dilution series. *Lentinula edodes* (Mori No. 290, Mori Sangyo Co., Ltd, Hokkaido, Japan) and *Pleurotus pulmonarius* (strain “Kanayama”) were grown at 25°C on potato dextrose agar plates for 2 weeks. After separate cultivation of each species in liquid potato dextrose medium for 1 to 2 weeks, mycelia were harvested, lyophilized, and ground into powder. Branches of *Quercus serrata* Murray (Japanese konara oak), which is one of the most popular bed log substrates for commercial cultivation of *L*. *edodes* in Japan, were obtained in the Fujisawa Experimental Forest (Fujisawa, Japan). Branches were dried at 70°C in an oven for 1 week, and wood dust was produced by using an electric drill. Wood dust was autoclaved (121°C, 1 h), lyophilized, and were ground to powder with a Multi-beads Shocker (Yasui Kikai Corporation, Osaka, Japan).

### DNA extraction

DNA was extracted in cetyltrimethylammonium bromide lysis buffer containing 0.6% skim milk using 10 mg of mycelia or plant material [20]. The extracted DNA was stored in sterile water at −20°C prior to use. The quality of the DNA samples was checked by 1.0% agarose gel (SeaKem GTG agarose; FMC BioProducts, Rockland, ME, USA) electrophoresis in a Tris-borate-EDTA buffer containing ethidium bromide.

### Polymerase chain reaction

Fungal and plant genomic DNA was amplified by PCR to verify extraction quality. PCR was conducted in 50-μL reaction mixtures containing 250 μM dNTPs, 300 nM NS1 and NS2 primers (universal primers for the small (18S) ribosomal subunit; [6]), 50 ng of template DNA, 1.0 unit of Takara Ex Taq (Takara Bio Co., Ltd., Shiga, Japan), and a universal buffer provided with the enzyme. PCR was performed in a GeneAmp 9700 PCR System (Applied Biosystems, Foster City, CA, USA) as follows: 1 cycle at 94°C for 2 min, followed by 30 cycles of denaturation at 94°C for 30 s, annealing at 55°C for 30 s, and elongation at 72°C for 7 min, and then cooling to 4°C for 20 min. Amplification products were electrophoresed on 2.0% agarose gels (a 3:2 ratio of NuSieve GTG agarose and SeaKem GTG agarose; FMC Bio Products, Tokyo, Japan) in Tris-borate-EDTA buffer containing 0.5 μg/mL ethidium bromide.

### Specificity of primers for *L*. *edodes*

Primers were designed based on the sequence of the gene encoding manganese peroxidase from *L*. *edodes (lemnp1*) [19]. A BLAST search of the publicly available *L*. *edodes* genome (http://forestgen.ffpri.affrc.go.jp/en/index.html) showed homology at only one position, indicating that this strain has only a single copy of *lemnp1*. From the 4460-bp DNA fragment, we designed a set of primers based on the sequences at bp 1595–1612 and bp 1807–1832, with the following sequences: Lek1f 5′- GGCTCATGAATCTCTGCG -3′ and Lek1r 5′- ATCGATACCACCTGTTGATATTTAAG -3′. To test the specificity of the primers for *L*. *edodes*, a qPCR analysis was performed using extracted DNA samples from all of the fungal and plant materials shown in Table 1 (*n* = 66) using a CFX96 analyzer (Bio-Rad Laboratories Inc., Hercules, CA, USA). DNA amplification and detection were performed in 100-μL PCR tubes in a total volume of 20 μL containing 10 μL of SYBR Green Realtime PCR Master Mix Plus (Toyobo Co., Ltd., Osaka, Japan), 2.0 μL (0.5 μM) of each primer, 7.0 μL of H_2_O (sterile PCR grade), and 1 μL of template DNA using the following program: 1 cycle at 95°C for 5 min, followed by 50 cycles at 95°C for 10 s, 60°C for 30 s, and 72°C for 30 s, followed by signal detection at 79°C. Melting curve analysis was conducted from 65°C to 95°C. PCR products with a single melting curve that fit the respective standard curve were considered to be authentic. The 237-bp fragments amplified by the Lek1f and Lek1r primers were electrophoresed in TAE 1.0% or 2.0% agarose gels.

### Standard curve for mycelial biomass versus plasmid copy number

Wood samples were adjusted to contain 10% powdered fungal mycelia that contained 0.1–10% *L*. *edodes* with the reminder composed of *P*. *pulmonarius* (see “Fungal and plant materials used for testing primer specificity” for details). To produce a standard curve for mycelial biomass versus the cycle threshold (Ct), DNA extracted from 10-mg wood samples that contained 0.01–1 mg of *L*. *edodes* was subjected to qPCR with the primers Lek1f and Lek1r. Specimens were diluted 1:100 prior to qPCR. The qPCR conditions were as described above (see “Specificity of primers for *L*. *edodes*”). To construct a standard curve for plasmid copy number versus fungal biomass, 237 bp of *L*. *edodes* target DNA were inserted into the pEX-A2J1 cloning vector (GenScript, Piscataway, NJ, USA). DNA copy number of samples was simultaneously calibrated using the standard curve for the plasmid pLek237.

To generate a standard curve for each qPCR, the known copy number of plasmid pLek237 was used as a reference for the stability and repeatability of fungal biomass quantification.

### Quantification of *L*. *edodes* biomass

To quantify the mycelial biomass of *L*. *edodes*, the copy number of the 237-bp DNA fragment in extracts from wood samples was analyzed by qPCR. The copy number of serial dilutions of pLek237 ranged from 10^7^ to 10^1^. The mycelial biomass of *L*. *edodes* was estimated from the copy number of the 237-bp DNA fragment using the standard curve of plasmid copy number versus fungal biomass.

### Validation via sampling of *L*. *edodes* inoculated on *Q*. *serrata* wood dust

To validate the accuracy of the method for quantification of *L*. *edodes*, we incubated wood dust with rice bran inoculated with *L*. *edodes* mycelia. Wood dust of *Q*. *serrata* was obtained from a living tree in Morioka, Iwate. We homogenized wood dust and rice bran at a ratio of 4 to 1 (dry weight). Aliquots of the wood dust and rice bran mixture (4 g, dry weight) were placed in 50 glass dishes of 9-cm diameter. After autoclaving (121°C, 60 min), sterilized water was added to each dish to a water content of 65%. Each dish was inoculated with a 9-mm-diameter sample of *L*. *edodes* (No. 290, Mori Corp. Ltd.,) mycelia grown on potato dextrose agar (Nissui, Tokyo, Japan) and covered with parafilm. We incubated the dishes at 25°C in a black box for 56 days. Samples were collected from each dish each week after measurement of respiration rate (see below). After lyophilizing for 48 h, sample dry weight was determined. Samples were ground to a powder with a Multi-beads Shocker (Yasui Kikai Corp., Osaka, Japan). DNA extraction and qPCR were performed as described above.

### Respiration rate

Respiration rate was measured each week at a clean bench. A glass dish without a lid was inserted into an acrylic chamber (volume: 2.2 L) and the CO_2_ concentration and humidity in the chamber were monitored by using an infrared gas analyzer (GMP343, Vaisala Inc., Vantaa, Finland) and hydrometer (RTR503, T and D Inc., Nagano, Japan). Sample surface temperature was measured using an infrared thermometer (561, Fluke Inc., Everett, WA, USA). Measurements were conducted for 5 min and data from the last 2 min were used to calculate respiration rate, to eliminate the effects of moisture exchange.

### Calculation of MMQ and growth rate

MMQ was calculated as follows,

$$MMQ=\frac{R(t)/{MW}_{CO2}∙{MW}_{c}/{MW}_{c}}{MB(t)∙MCC/{MW}_{c}∙10^{3}}$$

Where MMQ is microbial metabolic quotient (mmol C/mmol C biomass/h), R(*t*) is respiration rate at time *t* (mg CO_2_/kg/h), MW_CO2_ is molecular weight of CO_2_, MW_C_ is molecular weight of carbon, MB(*t*) is microbial biomass at time *t* (μg/g), MCC is microbial carbon content (0.44) [21], and *t* is measurement time. Growth rate was calculated as follows,

$$growth\;rate=\frac{MB(t+1)}{MB(t)}∙100.$$

### Statistical analysis

Pearson’s correlation analysis was used to examine the associations between microbial biomass, respiration rate, temperature, water content, log-transformed MMQ, and growth rate of microbial biomass. Values of *P* < 0.05 were considered statistically significant. All analyses were performed using R (version 3.5.2) [22].

## Results

### Validation of primer specificity for *L*. *edodes*

**Fig 1:**
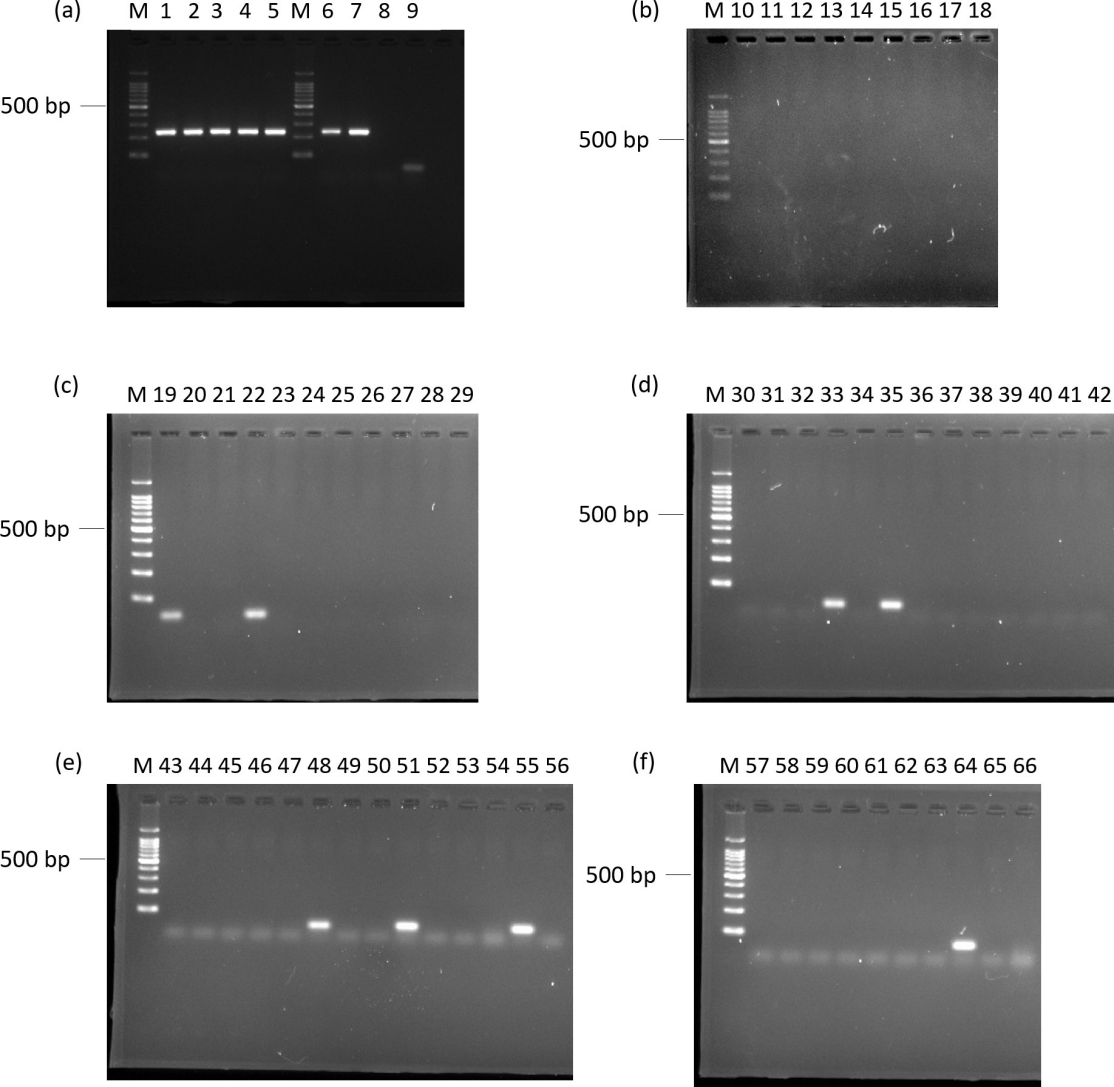
Agarose gel electrophoresis profiles of DNA fragments amplified by PCR. DNA from 66 fungal strains and plant materials was amplified using primers Lek1f and Lek1r: (a) Lanes 1–5, *Lentinula edodes* strains from Japan; Lines 6–9, *L*. *edodes* strains from Southeast Asia; (b) Lanes 10–18, related species; (c) Lanes 19–29, commercial species of fungi; (d) Lanes 30–42, saprotrophs; (e) Lanes 43–56, pathogens; (f) Lane 57–66, plant materials. Lane number corresponds to sample number in Table 1.

**Fig 2:**
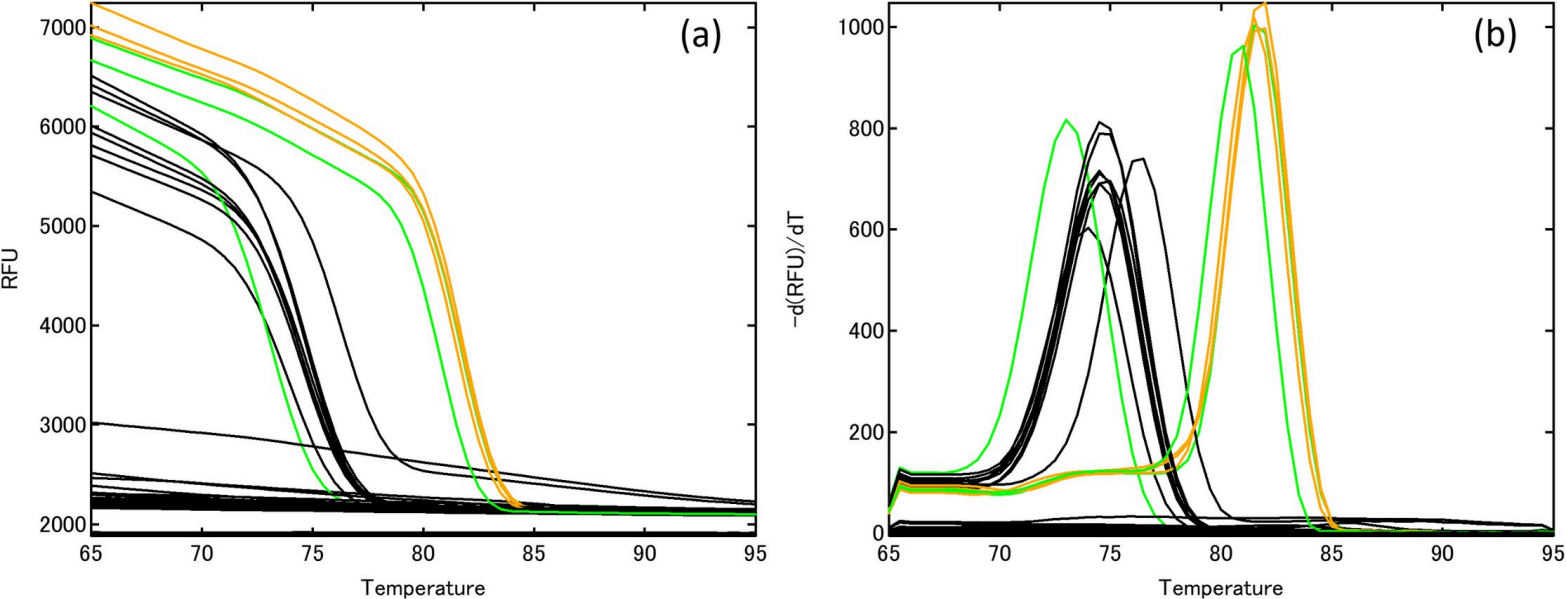
DNA melting curve analysis for 66 fungal strains and plant materials. (a) Derivative plots and (b) normalized melting curves. Orange lines, *Lentinula edodes* (Japan); green line, *L*. *edodes* (Southeast Asia, No. 8); black line, other strains (No. 19, 22, 33, 35, 47, 50, 55) and plant materials (No. 64). Numbers correspond to sample numbers in Table 1.

In order to validate the specificity of the primer pair Lek1f and Lek1r to *L*. *edodes*, agarose gel electrophoresis and melting curve analysis were conducted using qPCR. Amplification products, indicated by single bands of the anticipated size, were detected for all strains of *L*. *edodes* from Japan and for strains 6 and 7 from Indonesia and Papua New Guinea, respectively (Fig 1). Primer dimers or other non-specific amplification products were observed in one strain of *L*. *edodes* from Borneo (No. 9), two edible mushrooms (No. 19, 22), three pathogenic species (No. 48, 51, 55), two saprotrophs (No. 33, 35), and one tree species (No. 64). Melting curve analyses showed that all the genes derived from *L*. *edodes* and species that produced primer dimers had a single peak (Fig 2). The dissociation temperature for all amplified strains of *L*. *edodes* was 81.5–82°C; the other peaks had a lower dissociation temperature, ranging from 73 to 76.5°C, indicating that the primer pair can detect strains of *L*. *edodes* from Japan and closely related strains at dissociation temperatures higher than 76.5°C. Therefore, setting the signal detection temperature at 79°C can be used to test for Japanese and closely related strains of *L*. *edodes*.

### Quantification of fungal biomass of *L*. *edodes*

**Fig 3:**
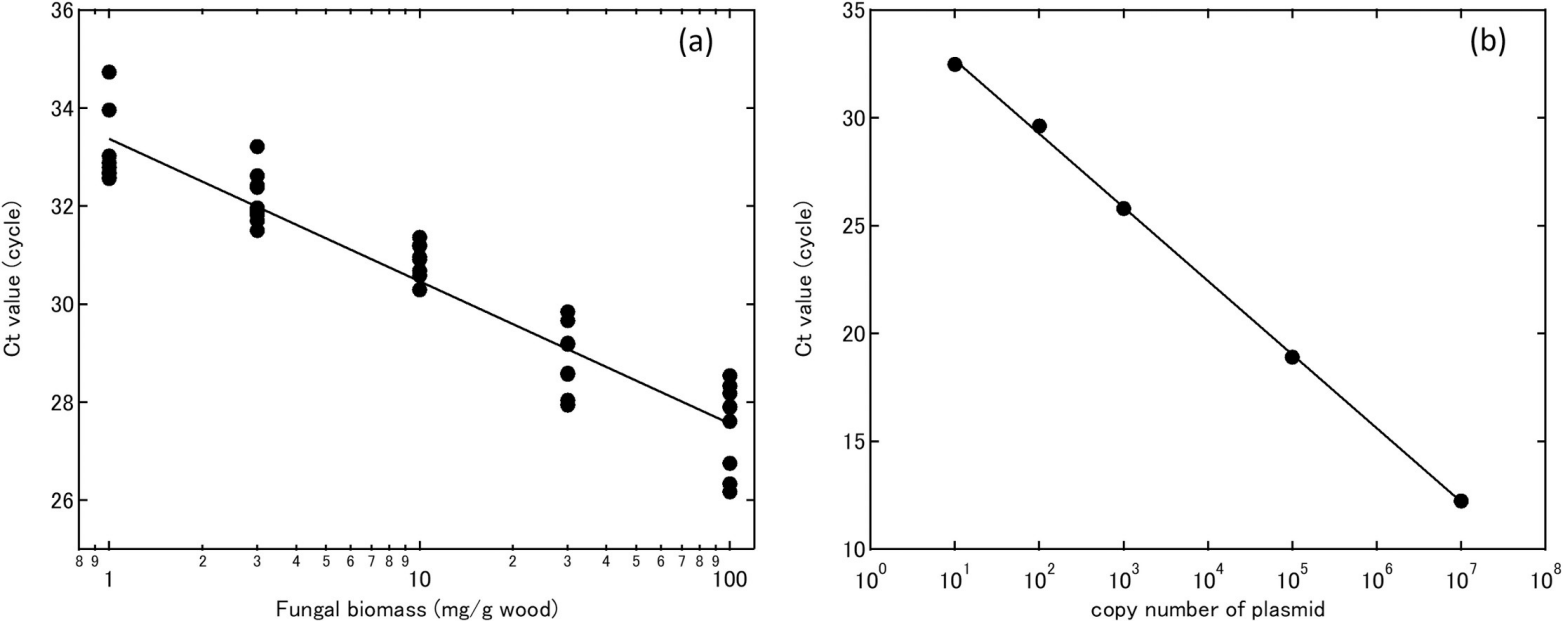
(a) Standard curve showing the relationship between cycle threshold (Ct) and *Lentinula edodes* biomass obtained using DNA amplicons extracted from *L*. *edodes* and *Quercus serrata* wood powder mix (0.1 to 10%). (b) Standard curve showing the relationship between Ct and copy number of plasmid pLek237 (10^1^ to 10^7^).

The standard curve generated for the wood–*L*. *edodes* mixed sample showed linearity between 1 and 1 × 10^2^ mg/g wood (Fig 3A). The standard curve generated for the plasmid showed linearity between 10^1^ to 10^7^ copy numbers μL^–1^ (Fig 3B).

### *Lentinula edodes* biomass and MMQ

**Fig 4:**
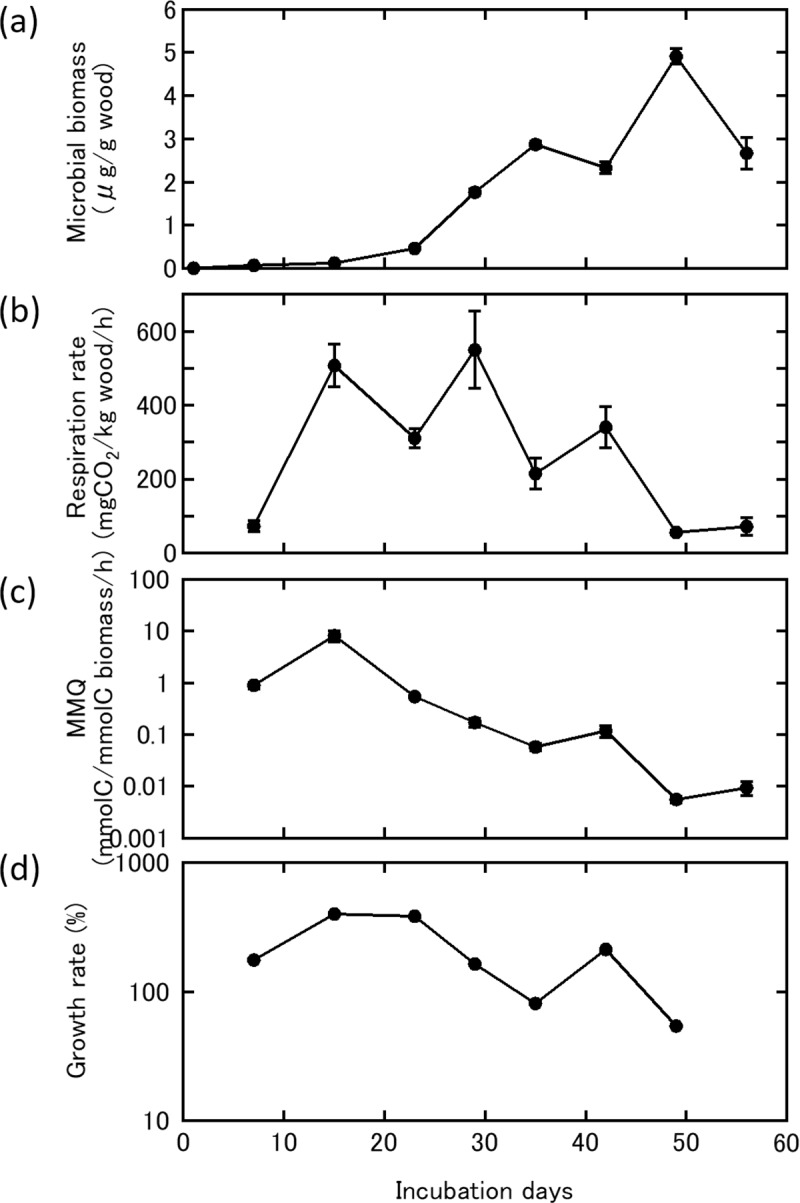
*Lentinula edodes* cultured on potato dextrose agar media was inoculated on *Quercus serrata* wood dust substrate mixed with 20% rice bran over 56 days at 25°C. (a) Microbial biomass of *Lentinula edodes* based on calculations from qPCR data; (b) respiration rate measured by infrared gas analyzer; (c) microbial metabolic quotient (MMQ) calculated by microbial biomass and respiration rate; and (d) growth rate based on the ratio of microbial biomass between two time intervals (*n* = 5, error bars show SE).

Biomass of *L*. *edodes* inoculated on wood dust and rice bran mixed media remained below the detection limit of 1 mg/g wood till 23 days of incubation, grew to 1.7 mg/g wood at 29 days of incubation, and reached a maximum of 4.9 mg/g wood at 49 days; mycelial biomass increased steadily during incubation, except for a small decrease between days 42 and 56 of incubation (Fig 4A). Respiration rate became detectable at 7 days (73.2 mg CO_2_/kg/h) and quickly increased to 520 mg CO_2_/kg/h after 15 days of incubation, decreased until day 23, then increased again to the maximum respiration rate of 549.8 mg CO_2_/kg/h reached at day 29 (Fig 4B). After reaching the maximum, respiration rate decreased to 71.8 mg CO_2_/kg/h after 49 days of incubation. MMQ was high in the early stages of incubation and decreased gradually toward the end of the incubation period. MMQ peaked at 8.1 nmol C/nmol C biomass/h on day 14 then decreased gradually, reaching a minimum of 0.0054 nmol C/nmol C biomass/h after 49 days of incubation (Fig 4C). Similarly, growth rate was high in the early stages of incubation and decreased with time (Fig 4D). Significant relationships were observed between log-transformed MMQ and microbial biomass (*P*<0.01), growth rate and mycelial biomass (*P*<0.05), and log-transformed MMQ and growth rate (*P*<0.05).

## Discussion

### Species specificity of *L*. *edodes* primers

*Lentinula edodes* has an Asian–Australian distribution [2, 22]. In this study, we used nine *L*. *edodes* strains, including five from Japan and four obtained in Cibodas, Indonesia, Papua New Guinea, and on Mt. Kinabalu, Borneo, Malaysia. The primer pair we designed amplified genes from all five strains from Japan and two of the four strains from Southeast Asia; the two strains from Borneo were not amplified. Wild *L*. *edodes* shows spatially distributed genetic variation and can be divided into five lineages based on rDNA data [23]. Hibbett et al. [23] showed that there are biogeographical differences in *L*. *edodes*, although wild isolates collected from Japan and Borneo can be categorized in the same group [23]. Because our aim was to develop primers for species-specific amplification of Japanese *L*. *edodes* strains, and our primer pair successfully amplified all five strains tested, the primer pair can be applied to biomass estimation for Japanese strains of *L*. *edodes*.

### Quantification of fungal biomass of *L*. *edodes*

We developed a qPCR procedure to estimate the fungal biomass of *L*. *edodes*, with primers specific to Japanese strains. Using a mixed sample of wood and a known amount of *L*. *edodes* mycelium, we were able to measure mycelial concentrations ≥1 mg/g wood. Using qPCR, we were able to quantify the increase in *L*. *edodes* biomass 1 day after the inoculation. These results suggest that the biomass of *L*. *edodes* growing in wood can be estimated using the developed qPCR procedure. Mycelial growth of *L*. *edodes* could be an important index for estimating the quality, quantity, and timing of mushroom production for commercial cultivation. Under natural conditions, various fungal species decompose dead wood, with each piece of dead wood potentially colonized by hundreds of fungal species, some of which show more than 90% relative abundance based on metagenomic analysis [9, 10]. Development of species-specific tools for estimation of biomass of dominant fungal species would be useful to determine the spatial and temporal patterns of species-level fungal biomass and growth rate under natural conditions.

The development of a species-specific method for estimation of *L*. *edodes* biomass enabled us to measure biomass and respiration rate simultaneously, and to monitor physiological activity per unit of biomass (MMQ). *Lentinula edodes* incubated on wood dust for 14 days exhibited a maximum MMQ of 8.1 nmol C/nmol C biomass/h. The maximum MMQ values measured here were higher than previously reported MMQ values, based on a meta-analysis lower than our MMQ values [18]. The high values of MMQ were caused by the high respiration rate of the small amount of fungal biomass. MMQ showed a similar pattern of increase and decrease to growth rate, and we hypothesize that fungi with a high MMQ in the early stages of incubation are preparing for mycelial growth and that MMQ is a useful indicator of later fungal growth. MMQ has been reported to be related to microbial stress and growth rate [12, 13, 15–17, 24]. Therefore, gaining a better understanding of the factors that influence MMQ would allow us to predict fungal biomass.

## Supporting information

S1 Table Raw data of fungal biomass and Ct values presented in Fig 3(A). (XLSX)

S2 Table Raw data of copy number of plasmid and Ct values presented in Fig 3(B). (XLSX)

S3 Table Data of the incubation experiment of Lentinula edodes inoculated on Quercus serrata wood dust substrate mixed with 20% rice bran over 56 days at 25°C. (XLSX)

S1 Raw Images (PNG)
