# Supplementary figures and images for: Mycelial biomass estimation and metabolic quotient of Lentinula edodes using species-specific qPCR
Source: PLoS One. 2020 May 18;15(5):e0232049. doi: 10.1371/journal.pone.0232049 (PMC7233531; doi:10.1371/journal.pone.0232049)

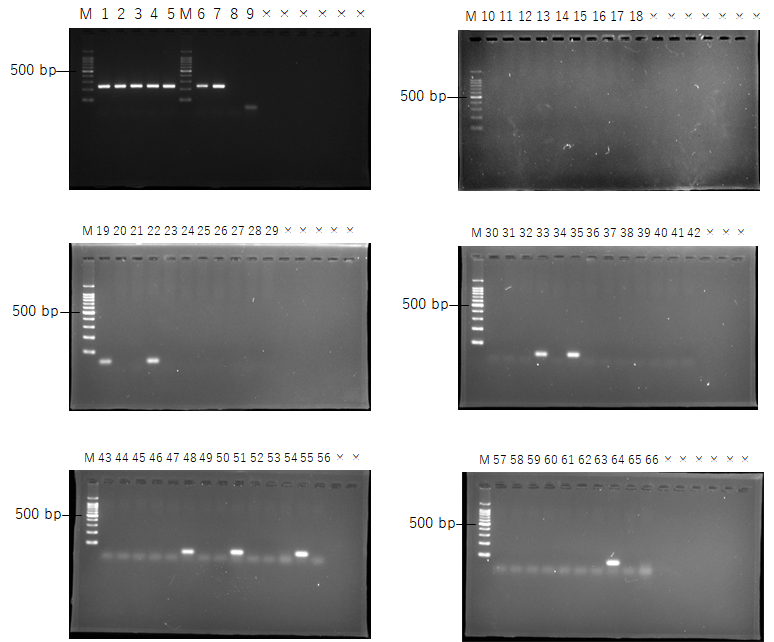

Supplement: S1 Raw Images — (PNG) [file pone.0232049.s004.png]
